# Spectrum of Hemorrhagic Encephalitis in COVID-19 Patients: A Case Series and Review

**DOI:** 10.3390/diagnostics12040924

**Published:** 2022-04-07

**Authors:** Rohan Sharma, Krishna Nalleballe, Vishank Shah, Shilpa Haldal, Thomas Spradley, Lana Hasan, Krishna Mylavarapu, Keyur Vyas, Manoj Kumar, Sanjeeva Onteddu, Murat Gokden, Nidhi Kapoor

**Affiliations:** 1 Department of Neurology, University of Arkansas for Medical Sciences, Little Rock, AR 72205, USA; rsharma2@uams.edu (R.S.); knalleballe@uams.edu (K.N.); shaldal@uams.edu (S.H.); sronteddu@uams.edu (S.O.); 2 Department of Neurology, John Hopkins University, Baltimore, MD 21205, USA; vshah21@jh.edu; 3 Department of Internal Medicine, University of Arkansas for Medical Sciences, Little Rock, AR 72205, USA; tpspradley@uams.edu (T.S.); lshasan@uams.edu (L.H.); 4 Baptist Health Medical Center, Little Rock, Little Rock, AR 72205, USA; chaitu1981@gmail.com; 5 Department of Infectious Diseases, University of Arkansas for Medical Sciences, Little Rock, AR 72205, USA; vyaskeyurs@uams.edu; 6 Department of Radiology, University of Arkansas for Medical Sciences, Little Rock, AR 72205, USA; mkumar@uams.edu; 7 Department of Pathology, University of Arkansas for Medical Sciences, Little Rock, AR 72205, USA; gokdenmurat@uams.edu

**Keywords:** COVID-19, hemorrhagic encephalitis, mucormycosis

## Abstract

Severe acute respiratory syndrome corona virus 2 (SARS-CoV-2) is an ongoing pandemic that has affected over 400 million people worldwide and caused nearly 6 million deaths. Hemorrhagic encephalitis is an uncommon but serious complication of COVID-19. The etiology of this disease is multifactorial, including secondary to severe hypoxemia, systemic inflammation, direct viral invasion, hypercoagulability, etc. The clinical spectrum of COVID-19-related hemorrhagic encephalitis is also varied, ranging from leukoencephalopathy with microhemorrhage, acute necrotizing hemorrhagic encephalitis (ANHE) involving the cortex, basal ganglia, rarely brain stem and cervical spine, hemorrhagic posterior reversible encephalopathy syndrome (PRES) to superimposed co-infection with other organisms. We report a case series of three young patients with different presentations of hemorrhagic encephalitis after COVID-19 infection and a review of the literature. One patient had self-limiting ANHE in the setting of mild COVID-19 systemic illness. The second patient had self-limiting leukoencephalopathy with microhemorrhages in the setting of severe systemic diseases and ARDS, and clinically improved with the resolution of systemic illness. Both patients were healthy and did not have any premorbid conditions. The third patient with poorly controlled diabetes and hypertension had severe systemic illness with neurological involvement including multiple ischemic strokes, basal meningitis, hemorrhagic encephalitis with pathological evidence of cerebral mucormycosis, and Epstein–Barr virus coinfection, and improved after antifungal therapy.

## 1. Introduction

COVID-19 is known to cause a myriad of neurological complications. Hemorrhagic encephalitis is one such uncommon but serious complication of COVID-19. The etiology of this disease is poorly understood, and the clinical spectrum of COVID-19-related hemorrhagic encephalitis is also varied. In this study we present three cases with different manifestations COVID-19-related hemorrhagic encephalitis and review of existing literature.

## 2. Case 1

A 43-year-old African American man without any significant past medical history presented with a new onset progressively worsening bitemporal headache over a week. He was initially admitted to an outside hospital (OSH) where he tested positive for severe acute respiratory syndrome corona virus 2 (SARS-CoV-2). Over the course of the next four days, he developed a fever with chills and was managed conservatively, after which he was discharged home. At home, he continued to have headaches. Two days after discharge he developed new-onset seizures, which were reported as generalized tonic–clonic in semiology and presented again to the outside hospital emergency department (ED). His vitals were reportedly stable, and a non-contrast CT head showed left temporal hypodensity. He was started on levetiracetam and transferred to our hospital for further management. On arrival his vitals were normal, he was awake and oriented to time, place, person, and situation. His neurological, respiratory, cardiovascular, and abdominal exams were also unremarkable. His initial labs were significant with a white blood cell count (WBC) of 15,470/mm^3^, hemoglobin (Hb) of 10.8 gm/dL, platelets count of 61,600/mm^3^, creatinine of 1.3 mg/dL, C-Reactive protein (CRP) of 42.4 mg/L, erythrocyte sedimentation rate (ESR) of 95 mm/h, creatine kinase (CK) 858 IU/L, D-Dimer of 1821 FEU, fibrinogen of 644 mg/dL, ferritin of 1352.9 ng/mL, lactate dehydrogenase (LD) of 392 IU/L, prothrombin time (PT/INR) of 1.3/15. The electrocardiogram (EKG) and chest X-ray (CXR) were unremarkable. A video electroencephalogram (VEEG) for 24 h was done for seizure workup and was also normal. An initial COVID-19 nasal polymerase chain reaction (PCR) on this admission was negative but a repeat test done 2 days later was positive.

**Figure 1:**
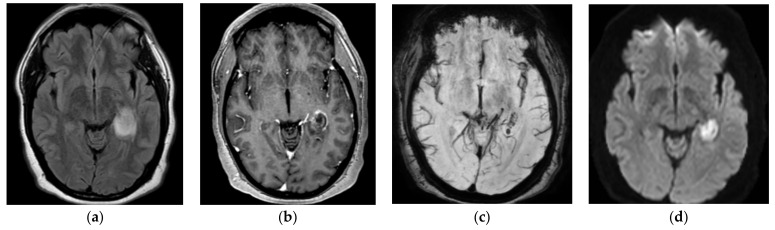
Hemorrhagic lesion in the left temporal lobe and parahippocampal gyrus. The lesion is hyper-intense on the FLAIR axial image (**a**). The lesion shows mild enhancement on the post-contrast T1 axial image (**b**). It shows a blooming artifact on susceptibility-weighted imaging (SWI) (**c**) image. The lesion shows restricted diffusion on the diffusion b 1000 (**d**).

MRI brain images with and without contrast were obtained in light of the new-onset seizures and temporal hypodensity on CT, which showed intense focal edema within the left hippocampus with mild restricted diffusion, postcontrast enhancement, and hemorrhage seen on susceptibility-weighted imaging (Figure 1). MRA head and neck images did not show any large vessel occlusion or flow-limiting stenosis. The patient was started on empiric cefepime and vancomycin for concerns of meningoencephalitis given the MRI results, and extensive infectious workup was done to assess for the underlying infectious agent. Cerebrospinal fluid (CSF) studies revealed a leucocyte count of 57/μL with neutrophilic predominance (neutrophils: 50%, lymphocytes: 28%, monocytes: 22%), protein of 44 mg/dL, and glucose 59 mg/dL. The CSF gram stain, bacterial culture, and meningitis/encephalitis panel (including cytomegalovirus, enterovirus, *E. coli* K1, Hemophilus influenzae, herpessSimplex virus (HSV)-1, and HSV 2, human herpes virus 6, Listeria, *Neisseria meningitidis*, parechovirus, *Streptococcus agalactiae*, *Streptococcus pneumoniae*) were negative. Varicella zoster virus (VZV) PCR, cryptococcal antigen, culture, acid fast bacilli (AFB) smear and culture, and *Cytomegalovirus* (CMV) PCR were negative. CSF VZV IgG were found to be greater than 4000 IU. Infectious disease (ID) was consulted and vancomycin and cefepime were continued for a total of 14 days as per their recommendation for concern of encephalitis due to neutrophilic pleocytosis in CSF. The patient’s clinical course was stable during the course of admission. He did not have any further seizure episodes. He was discharged home on the tenth day of admission. He continued to have occasional headaches after discharge but otherwise was completely back to baseline. An MRI brain scan done 7 weeks later showed a persistent susceptibility artifact, suggestive of hemorrhage in the left parahippocampal gyrus with a resolution of adjacent enhancement and mass effect with interval volume loss. Overall, he manifested self-limiting hemorrhagic encephalitis in the setting of mild systemic COVID-19 disease.

## 3. Case 2

A 43-year-old Hispanic man without any significant past medical history presented with a history of subjective fever, cough, sore throat, chest tightness, pain in the lower chest with deep breaths, and worsening shortness of breath over the past 10 days which worsened with exertion. In ED, he was diaphoretic, normotensive, and tachypneic, with oxygen saturation in the 70s on room air, which improved to the low 90s on 50% supplemental oxygen. He had decreased breath sounds and rales present in bilateral lung fields and the rest of the physical exam was unremarkable. His chest X-ray showed bilateral florid multifocal infiltrates and his EKG was unremarkable. He was admitted to ICU for acute hypoxemic respiratory failure. His labs were significant with a WBC count of 9940/mm^3^, platelet count of 68,000/mm^3^, interleukin-6 of 32 pg/mL, ferritin of 1092 ng/mL, CRP of 139.9 mg/L, and fibrinogen 781 mg/dL. The patient was started on vancomycin, ceftriaxone, and azithromycin for concerns of concomitant bacterial pneumonia and dexamethasone for concerns of ARDS in the setting of COVID-19 pneumonia.

The patient was later transitioned to noninvasive Bilevel positive airway pressure (BiPap) ventilation on the same day for worsening hypoxemia and his azithromycin was changed to doxycycline due to a prolonged QTc interval. Vancomycin was discontinued after the nasal MRSA PCR was negative. He continued to worsen over the next day and was intubated on the third day of admission for acute hypoxemic hypercapnic respiratory failure. His SARS-CoV-2 nasal PCR was negative twice, and a bronchoalveolar lavage (BAL) was sent for SARS-CoV-2 PCR, which later turned out to be positive. The patient also required pressors due to hypotension and also developed a small pneumothorax likely as a complication of central venous line placement. The patient became agitated and self-extubated on the fifth day but continued to be hypoxemic requiring reintubation. The patient later developed acute respiratory distress syndrome (ARDS) requiring a high inhaled oxygen fraction and positive end-expiratory pressure. He continued to have elevated IL-6, fibrinogen, and thrombocytopenia. The patient was also started on remdesivir for COVID-19 pneumonia. The patient completed a course of ceftriaxone and doxycycline for community-acquired pneumonia (CAP), a 10-day course of dexamethasone, a 10-day course of remdesivir, and received convalescent plasma twice. He developed a fever on the twelfth day and was started on cefepime for a fever which was transitioned to meropenem for 7 days as his tracheal aspirate grew beta-lactam-resistant Klebsiella pneumonia. The patient continued to be encephalopathic and agitated and it was difficult to wean from sedation due to elevated blood pressures and tachycardia with weaning trials; however, sedation was eventually weaned with clonidine, oxycodone, and quetiapine and he was extubated on day 21. The patient still had an increased oxygen requirement requiring a venti mask. He was transferred to the floor on day 25.

**Figure 2:**
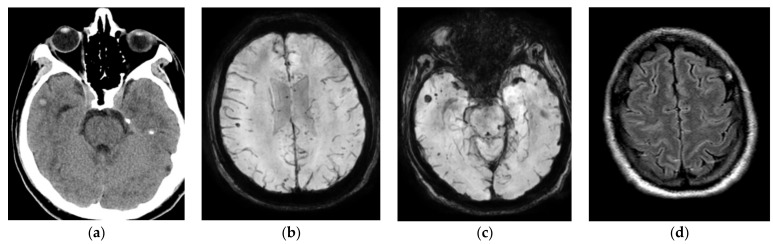
Multiple hemorrhages in the supratentorium. CTH without contrast showing right temporal hyperdensity suggestive of hemorrhage (**a**). MRI of the brain with and without showed small right anterior temporal lobe intraparenchymal hemorrhage (**b**); additional multiple scattered foci of susceptibility artifact particularly in the gray–white junctions and corpus callosum (**c**); and sulcal FLAIR hyperintensity in the right frontal, biparietal, and left temporal lobes (**d**).

On day 26, the patient became more encephalopathic, became non-verbal, and had an episode of rapid eye fluttering and gaze deviation, and neurology was consulted for concerns of stroke/seizures. Upon examination, the patient was diaphoretic, opening eyes spontaneously, mute but following simple commands, and moving all four extremities spontaneously. A stat CT head and CT angiogram head and neck were obtained for concerns of acute stroke which showed a small right temporal hyperdensity (0.6 cm diameter) suggestive of a hemorrhage with normal vasculature (Figure 2). The patient was started on Levetiracetam and transferred to ICU for closer monitoring. An MRI of the brain with and without was obtained in light of the acute hemorrhage which showed a stable small right anterior temporal lobe intraparenchymal hemorrhage, additional multiple scattered foci of susceptibility artifact, particularly in the gray–white junctions and corpus callosum, and sulcal FLAIR hyperintensity in the right frontal, biparietal, and left temporal lobes without any postcontrast enhancement (Figure 2). The patient was started on VEEG for 24 h for new-onset status epilepticus which showed generalized slowing without any ictal or inter-ictal discharges. CSF studies showed glucose of 28 mg/dL, protein of 118 mg/dL, xanthochromia with 91,000/μL RBCs, 270/μL WBCs with neutrophilic predominance (80%). Other CSF studies including Meningitis/encephalitis panel, AFB smear and culture, cryptococcal antigen, and VZV PCR, CMV PCR, CSF cytology were negative. The patient’s mental status improved with levetiracetam and he was transferred to the floor on day 29. His mental status continued to improve to the point that he returned back to his normal self and was discharged on day 32 with levetiracetam for 2 weeks. He was seen in the clinic after 1 month and did not have any seizures but complained of recurrent headaches. His exam was unremarkable.

## 4. Case 3

A 52-year-old Hispanic man with a past medical history of hypertension, diabetes mellitus, and hyperlipidemia presented with a worsening cough, shortness of breath, and fever for a week. The patient was found to be positive for SARS-CoV-2 on nasal PCR. The patient was initially treated as an outpatient but presented to an outside hospital 3 weeks later with a progressively worsening left-sided headache that radiated to the back of the left eye, associated with blurred vision for 1 week, not associated with any nausea or vomiting. He also developed sudden onset left-sided ptosis on the day prior to presentation. He did not report any neck stiffness, confusion, photophobia, fever, chills, focal weakness, numbness, tingling, slurred speech, or facial drooping. His exam was reportedly unremarkable except for left-sided ptosis, restricted extraocular movements, and tenderness over the left temporal area. The labs were significant with a WBC of 12,000/μL, serum glucose of 412 mg/dL, creatinine of 1.4 mg/dL, ESR 111 mm/h, CRP 43.5 mg/L, and HbA1c 12.6%. A CT angiogram of the head and neck did not show any evidence of stroke, hemorrhage, or any large vessel occlusion, but did reveal stenosis of the cavernous segment of the right ICA and patchy infiltrates in bilateral lung fields. An MRI of the brain and MR venogram with and without contrast was subsequently done which showed mild periventricular white matter disease but were otherwise unremarkable. The patient was started on high-dose IV steroids for concerns of temporal arteritis for 3 days, and a left temporal artery biopsy was done which did not show any evidence of temporal arteritis, and the steroids were discontinued. The patient was diagnosed with ischemic third and sixth nerve palsy related to COVID-19/diabetes and hypertension, and discharged home on the fifth day of admission on a new insulin regimen.

**Figure 3:**
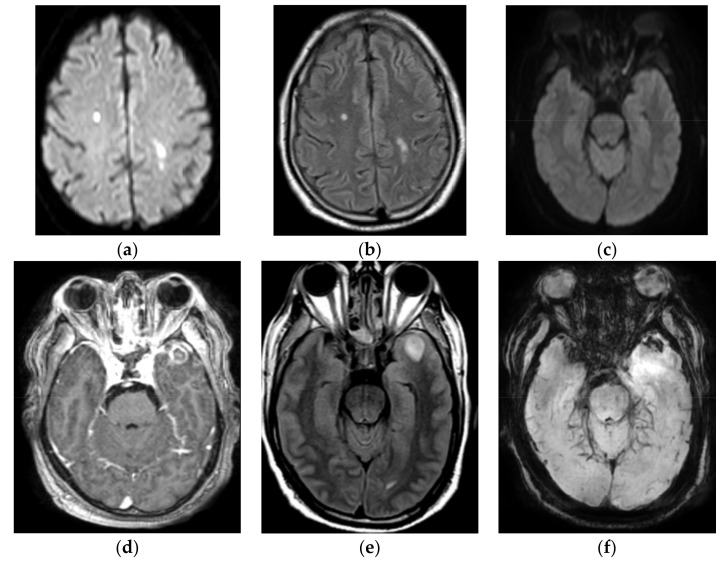
(**a**–**c**) Multiple acute infarcts. Multiple areas of restricting diffusion involving the right frontal and the left posterior frontal lobes on the b 1000 (**a**). Corresponding hyperintensities on FLAIR images (**b**). Restricted diffusion involving the left optic nerve on the b1000 (**c**). (**d**–**f**) Abscess in the left anterior temporal lobe. Peripherally enhancing lesion in the left anterior temporal lobe is seen on the post-contrast axial T1 image (**d**). Corresponding hyperintense signal on the FLAIR image (**e**) and susceptibility artifact on SWI images (**f**).

The patient returned to the OSH ED with sudden right hemiparesis and right eye ptosis. He also reportedly continued to have a severe bifrontal headache after discharge. Upon presentation, his pulse oximetry showed hypoxemia (90%), and other vitals were normal. The physical exam was significant for chemosis and conjunctival injection with bilateral ptosis, a dilated, and non-reactive pupil in the left eye, normally reactive pupil in the right eye with visual acuity decreased to finger counting on the left and 20/70 in right, and right upper and lower extremity weakness 4/5 proximally and distally. The rest of the neurological exam was reportedly unremarkable. The patient’s labs showed WBC of 13 k/μL, blood glucose 306 mg/dL, and CRP 295 mg/L. His PT/INR, urine analysis, blood culture, urine toxicology screen, urine electrolytes, HIV screen, ANA panel, liver, and renal function were unremarkable. A repeat CT head and CTA head and neck were unchanged from the prior study but the MRI brain scan with and without contrast revealed multiple punctate foci of restricted diffusion in the bilateral frontal, parietal, occipital, and temporal lobes, mild meningeal enhancement of the anterior and middle cranial fossa and opacification of multiple ethmoid and mastoid air cells (Figure 3). An MRI orbit with and without contrast was normal. EKG and transthoracic echo with bubble study were unremarkable. The patient was tested again for SARS-CoV-2 and was found to be positive on nasal PCR. CSF studies revealed WBC of 272/μL with lymphocytic predominance (neutrophils: 40%, lymphocytes: 57%), RBC of 674/μL, protein of 82 mg/dL, glucose 78 mg/dL, *Borrelia burgdorferi* IgM/IgG titer (0.09 LIV, 0.09 LIV), negative CSF ACE, VDRL, and cryptococcal antigen. He was started on vancomycin and ceftriaxone for concern of meningitis and was also started on steroids and a CSF autoimmune encephalitis panel was sent to Mayo Clinic. His CSF culture and encephalitis panel were negative but the serum hypercoagulation panel was positive for anti-cardiolipin IgM antibody titers of 27 MPL U/mL. The patient was started on aspirin and therapeutic enoxaparin and transferred to our hospital on day 5 of admission for further workup and management.

On arrival at our hospital, the patient’s vitals were stable and the clinical exam was significant for chemosis and conjunctival injection, bilateral ptosis, complete ophthalmoplegia in the left eye, near-complete ophthalmoplegia in the right eye with minimal abduction, dilated 6 mm and a non-reactive pupil in the left eye with complete loss of vision, and a 3-mm sluggishly reactive pupil with visual acuity of 20/40 and intact visual fields in the right eye. The rest of the ocular exam including cornea, iris, anterior chamber, posterior chamber, retina, optic disc, and retinal arterial and venous vessels were completely normal. The patient had decreased sensations on right V2 distribution. The rest of the systemic and neurological exam was normal. Labs were significant for WBC of 17 k/μL, blood glucose of 415mg/dL, fibrinogen of >1000 mg/dL, ferritin of 743 ng/mL, ESR of >130 mm/h, CRP of 313 mg/L, PT/INR of 19/1.6, procalcitonin of 1.24 ng/mL, and antithrombin-3 activity of 62%. His SARS-CoV-2 nasal PCR was negative. Extensive workup was done to assess for possible AIDP, myasthenia gravis, or vasculitis, and secondary infections, including the LFT, UA, ANCA panels, ANA panel, myasthenia panel, ganglioside panel, T-Spot TB test, urine histoplasma, blastomyces antigen, mycoplasma IgM (mycoplasma IgG was 0.81 U/L), anti-cardiolipin antibodies, B-2 glycoprotein, anti-Xa essay, HIV antibodies, hepatitis panel, serum cryoglobulin PAVAL autoimmune encephalitis panel, were negative.

A repeat MRI brain with and without contrast with MRA head and neck showed stable areas of punctate restricted diffusion in the bilateral frontal, parietal, occipital, and temporal lobes, mild linear enhancement seen in the dura overlying the bilateral anterior, and medial temporal lobes without any significant occlusion of major vessels. MRI orbit with and without contrast was obtained 2 days later due to clinical worsening upon the ophthalmological exam, which showed restricted diffusion along the mid-to-posterior left optic nerve with a focal area of T2 hyperintensity on the coronal images and a small focus of possible postcontrast enhancement in the retro-bulbar segment. It also showed a new 1.2-cm left anterior temporal hemorrhagic lesion with peripheral enhancement (Figure 3).

A repeat LP was obtained which showed an opening pressure of 18.5, and CSF studies showed pleocytosis 173/μL with lymphocytic predominance (neutrophils: 24%, lymphocytes: 63%), 6 RBCs, protein of 62 mg/dL, glucose of 75 mg/dL, and a CSF glucose/serum glucose ratio of 0.54. In the interim, he became hypoxemic and hypotensive and was started on vancomycin and cefepime along with IV fluids and supplemental oxygen and was transferred to ICU. CSF studies were significant for EBV PCR (positive in both CSF and plasma) and CSF IgG of 9.6 mg/dL. At this point in time a battery of test was done as the patient’s syndrome could not be defined and the etiology remained elusive: the Gram stain, acid fast staining, bacterial and fungal culture, infectious encephalitis panel, autoimmune encephalitis panel (ENC2 sent at OSH), anti-NMO/anti-MOG antibodies (CSF and serum), oncologic cytology, VDRL/RPR, ACE, Lyme antibodies, West Nile virus antibodies, SARS-CoV-2 PCR, VZV PCR, CMV PCR, TB PCR, and flow cytometry were negative. Blood cultures and serum B-Glucan and galactomannan were also negative. The patient was started on plasmapheresis for concern of IgG4-related autoimmune/inflammatory encephalitis which can cause similar presentation including basal meningitis, strokes, and encephalopathy. Vancomycin and cefepime were discontinued after 7 days.

The patient continued to have worsening encephalopathy and a repeat MRI brain scan with thin cuts through the skull base revealed extensive dural enhancement along the bilateral anterior and medial temporal lobes extending to the cavernous sinus, with a subtle extension into the bilateral tentorium and evolution of prior ischemic infarcts and a left temporal hemorrhagic lesion with peripheral enhancement and vasogenic edema. A repeat LP was done which showed RBCs of 651/μL, neutrophilic pleocytosis 195/μL (neutrophils: 60%, lymphocytes: 30%), protein of 90 mg/dL, and glucose of 37 mg/dL. Repeat CSF culture, encephalitis panel, SARS-CoV-2 PCR, and TB PCR were negative. The patient had undergone 2 cycles of plasmapheresis which was then discontinued, and the patient was started on IV Amphotericin B on the tenth day of admission for concern of possible fungal encephalitis in the setting of basal meningitis with possible cavernous sinus involvement. VEEG showed mild generalized slowing without any ictal or inter-ictal discharges. The patient also had a digital subtraction angiogram which was unremarkable. A CT maxillofacial was done to assess for sinus fungal infection which showed mucosal thickening involving the ethmoid, maxillary, and sphenoid sinuses without any osseous erosion/destruction. A nasal endoscopy was done by ENT which was also unremarkable and nasopharyngeal drainage was sent to culture. Amphotericin B was discontinued based on the recommendation of ID specialists.

**Figure 4:**
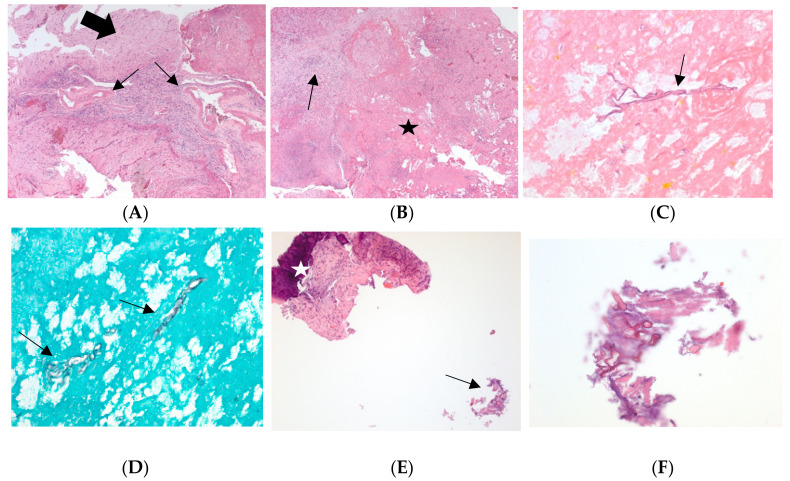
(**A**) 40× Granulation tissue with chronic inflammation fills and expands the subarachnoid space in a sulcus between two gyri (arrows), entrapping blood vessels (black arrow). (**B**) 40× The parenchymal lesion with areas of necrosis (star) surrounded by inflamed granulation tissue (arrows) and neuroglial parenchyma with reactive changes (left upper corner). (**C**) 400× Fungal hyphae (arrows) within the necrosis. (**D**) 400× Fungal hyphae (arrows) further highlighted by GMS stain. (**E**) 100× Curetting of sphenoid bone with aggregates of fungal hyphae (arrow) along with bone trabecula (star) surrounded by inflamed granulation tissue. (**F**) 400× Aggregation of fungal hyphae in sphenoid bone (hematoxylin and eosin unless otherwise specified).

On the twelfth day, the patient’s clinical condition worsened, and he became hypotensive and tachypneic, requiring IV fluids, pressors, and intubation for respiratory distress. Vancomycin, ceftriaxone, and doxycycline were restarted for concerns of sepsis. CT chest showed redemonstration of bilateral subpleural bands and patchy ground-glass opacities in both lungs, greater in the lung bases, and grossly unchanged from prior imaging (lung opacities in prior CTA neck). Given that the etiology of the patient’s meningitis remained unknown, a brain biopsy was done on the thirteenth day of admission and preliminary studies revealed necro-hemorrhagic tissues with acute and chronic inflammation and fungal hyphae, and the patient was restarted on amphotericin B. The patient’s clinical condition continued to deteriorate and his antibiotics were changed to vancomycin, meropenem, and amphotericin B, and IV isavuconazonium was added in light of the presence of fungal hyphae in sphenoid bone pathology. The pathology report showed scattered fungal hyphae consistent with Mucor species on all specimen parts including sphenoid bone, but not on dura mater (Figure 4). Additionally, scattered cells in temporal lobe biopsy specimens were found to be positive for EBV (Figure 4). A repeat MRI brain with MRA and MRV with contrast showed postoperative findings after resection of the ring-enhancing lesion in the left temporal lobe, extensive dural-based and diffuse skull base enhancement, with unremarkable MRA and MRV. Neurosurgery and ENT were consulted for possible surgical intervention but did not deem the patient a candidate for surgery given the extensive skull-based involvement on MRI. The patient was continued on antibiotics and dual antifungals.

The patient’s clinical condition improved, and he was extubated. After a discussion with the patient’s family, hiscode status was changed to DNR/DNI, and medical management was continued. The patient continued to improve with medical management and was transferred to the floor. He completed amphotericin B for 14 days and was continued on isavuconazonium orally for life as per ID’s recommendations. A stability MRI brain with and without contrast was repeated on day 41 of admission prior to discharge, showing stable post-contrast enhancement around the surgical site and dural enhancement around the middle cranial fossa and skull base. His neurological exam improved to the point that he was able to ambulate with assistance. He had no light perception in the left eye with complete ophthalmoplegia, intact vision in the right eye with ptosis, and near-complete ophthalmoplegia and preserved abduction. He was discharged on the forty-fifth day of admission.

## 5. Discussion

Severe acute respiratory syndrome corona virus 2 (SARS-CoV-2) was first identified in Wuhan, China in December 2019 [1], and has since caused a pandemic affecting over 200 million people worldwide with over 4.5 million deaths [2]. A myriad of neuropsychiatric manifestations of COVID-19 has been reported including ischemic strokes, seizures, encephalopathy, headaches, sleep disorders, polyneuropathy, myalgias, mood disorder, and anxiety and depression [3,4,5,6,7,8]. COVID-19-related acute encephalopathy is multifactorial, most often related to underlying systemic inflammation, ischemic strokes, or hypoxic injury, and is associated with increased mortality [9]. Rarer causes of encephalopathy, namely viral encephalitis [10,11,12], leukoencephalopathy, acute necrotizing hemorrhagic encephalitis (HE) [13,14,15,16,17], and autoimmune brain stem encephalitis [17,18] have all been reported as complications of COVID-19.

The overall rate of encephalitis in COVID-19 is less than 1% but can be as high as 6–7% in severely ill patients and typically presents in 2 weeks from the onset of symptoms [19]. The clinical signs and symptoms are similar to other causes of encephalitis, such as an impaired level of consciousness, seizures, headache, etc., but the mortality rate was found to be about 13% in a meta-analysis [19], which is considerably high as compared to other encephalitides [20,21]. This may be due to the multi-organ dysfunction and a lack of definitive therapy, at least in the initial stages of the pandemic. Encephalitis may present with meningeal, parenchymal, and/or white matter inflammation and in rare cases hemorrhagic encephalitis [22]. HE secondary to COVID-19 is rare but has a wide spectrum in terms of clinical and radiological features. The most common presentation is diffuse leukoencephalopathy with cortical microhemorrhage which is often seen in the setting of severe respiratory illnesses such as ARDS [16,23,24]. The microhemorrhages are typically seen in the corpus callosum, juxtacortical U-fibrers, and other large white matter tracts [22]. These microhemorrhages are typically CT negative and best seen on susceptibility-weighted sequences (SWI) on MRI and may produce T2 hyperintensities if a large number of confluent microhemorrhages are present [22]. This clinical picture can be seen in the setting of critical illness, severe hypoxemia, and ARDS due to causes other than COVID-19 [23,25]. Another subtype is acute necrotizing hemorrhagic encephalitis (ANHE) involving the temporal lobes similar to that of herpes, but hemorrhage in other lobes and subcortex can also be seen [14,26,27]. These lesions are often ring-enhancing with surrounding vasogenic edema and may also show diffusion restriction on MR imaging [13]. These patients are severely ill, and it is often fatal. Another subtype is hemorrhagic reversible posterior reversible encephalopathy (PRES) which manifests with vasogenic edema seen on MRI in parietal and occipital lobes typical of PRES, with cortical hemorrhage seen on SWI imaging [28,29]. PRES is also rarely seen in COVID-19 patients, typically in the setting of severe systemic illness [29,30]. The clinical prognosis of these patients is more favorable than other subtypes of COCID-19 HE. There have also been rare cases of COVID-19-related hemorrhagic rhombencephalitis [15,31] and myelitis [15,32] manifested by hemorrhagic lesions in the brain stem or upper cervical spinal cord. The imaging findings are similar to those of ANHE but isolated only to the brain stem or spinal cord.

The exact mechanism of COVID-19 HE is unknown, but may be multifactorial. Direct viral neuroinvasion [33] by SARS-CoV-2 has been proposed with limited evidence, although neuroinvasion has been reported with SARS-CoV-1 and other coronaviruses [34,35]. Other neurotropic viruses such as EBV and HSV are also known to cause HE [36,37,38]. Matschke et al. found SARS-CoV-2 in brain autopsy in half of the patients who died of COVID-19, but the presence of SARS-CoV-2 in the CNS was not associated with the severity of neuropathological changes [39]. This suggests that inflammatory processes, particularly cytokine-mediated CNS inflammation, may be more important [40,41]. Similar cytokine-mediated hemorrhagic encephalitides have been described with other viral infections such as influenza A and B, enterovirus, dengue, etc. [38,42,43,44,45]. Additionally, inflammatory disruption of the blood–brain barrier by endothelial cell activation is also proposed as a mechanism of vascular and brain injury [23,46], which can lead to vasogenic edema and PRES. Other possible mechanisms include hypoxemia, particularly in COVID-19 patients with ARDS, which is associated with cerebral micro-hemorrhages in the subcortical white matter and corpus callosum [23]. Additionally, COVID-19-related coagulopathy may lead to thrombosis in small medullary veins, leading to cerebral micro-hemorrhages [22,46]. These micro-hemorrhages may evolve into necrotic hemorrhagic lesions.

Finally, superimposed secondary infections, particularly in patients with co-morbidities, may be a contributor. We reported three young patients with HE after COVID-19 infection, of which two had a self-limiting disease that improved with the resolution of the systemic illness, and one had pathological evidence of fungal and viral invasion and improved only after antifungal therapy.

The first patient described had no co-morbidities and presented with mild COVID-19 systemic disease, and consequently, the encephalitis was self-limiting as well. His initial neurologic symptom was a headache, which is relatively common in COVID-19 patients [3,4,6]. Subsequently, he presented with a seizure, and neuroimaging showed a small necrotizing hemorrhagic lesion in the parahippocampal gyrus. His neurological examination was normal likely due to hemorrhage in the non-eloquent part of the temporal lobe. Based on the clinical picture and neuroimaging, this patient likely had ANHE, possibly as a consequence of direct neural invasion, although we were unable to prove this due to lack of availability of CSF SARS-CoV-2 PCR testing at the time, and given that the patient was neurologically intact, we did not pursue brain biopsy. Regardless, all other viral PCRs, bacterial, fungal, and mycobacterial cultures were negative, ruling out other infectious processes. The patient did have elevated VZV IgG titer in the CSF. Thus, co-infection or reactivation of VZV could not be excluded, but given that the clinical course improved without anti-viral therapy and the VZV PCR was negative, this was thought to be less likely. Additionally, IL-6 was not elevated, and systemic COVID-19 illness was not severe, both of which would have been expected in cytokine-mediated HE. Finally, the lesion was located in and limited to the parahippocampal region. Olfactory bulb projections to parahippocampal gyri have been described [47,48], which is suggestive of CNS invasion [49].

Contrarily, the second patient, who also had no comorbidities, presented with severe COVID-19 disease complicated by ARDS requiring prolonged mechanical ventilation and ICU stay. Neurologically, the patient presented with severe encephalopathy and recurrent seizures. Neuroimaging showed multiple supratentorial cerebral microhemorrhages and a larger hemorrhagic lesion in the temporal lobe. Marked elevation of IL-6 and other inflammatory mediators was noted. His clinical history and exam were consistent with leukoencephalopathy with microhemorrhages as a consequence of cytokine-mediated inflammation. A larger focal lesion in the temporal lobe does raise the possibility of viral neuroinvasion, but CSF COVID-19 testing was not available at this time and a brain biopsy was not pursued as the patient improved clinically. Although the patient had severe neurologic symptoms, they resolved with improvement in systemic COVID-19 disease, and at the time of discharge, the patient was at his premorbid neurologic baseline.

The third patient had uncontrolled type 2 diabetes and even though COVID-19 systemic disease was moderate, he presented with a headache, encephalopathy, and multiple cranial neuropathies three weeks after COVID-19 infection. He had multiple embolic strokes, ophthalmoplegia with hemorrhagic encephalitis involving the temporal lobe, multiple cranial neuropathies, and coagulopathy. These constellations of symptoms can be seen in the setting of COVID-19 [4,8,10,13,50]. Extensive workup was done to assess for other infections, vasculitis, or autoimmune disorders including neurosarcoidosis and IgG4-related encephalitis, but the testing was negative. The patient was empirically treated with plasmapheresis which was discontinued after clinical worsening and a new hemorrhagic lesion on MRI. CNS fungal infection was very low on the differential for this patient. Rhinocerebral mucormycosis has a very poor prognosis with a mortality rate of as high as 85% and requires frequent surgical debridement [51] but he had indolent rhinocerebral mucormycosis and survived for several weeks without antifungals. In addition, there was no evidence of locally invasive disease on brain/sinus imaging, and workup for fungal infections including nasal endoscopy and fungal culture was negative and the diagnosis was eventually made based on brain biopsy. His brain biopsy was also positive for EBV which is also known to cause hemorrhagic encephalitis also known as Weston–Hurst syndrome, or Hurst disease [52,53,54]. His encephalitis was very severe, and his symptoms likely emanated from a combination of the COVID-19 disease spectrum, rhinocerebral mucormycosis, and EBV encephalitis which made the clinical picture very confusing.

Coinfections with bacteria and fungi have been reported with COVID-19 disease [55] and may also occur in the central nervous system, leading to HE secondary to invasive mucormycosis in the setting of COVID-19 infection, which has been described previously [56,57,58,59], especially in the setting of hyperglycemia. It is unclear if COVID-19 infection directly contributes to the development of invasive fungal disease but may predispose due to leukopenia, lymphopenia, worsening hyperglycemia [60], and use of glucocorticoids, all factors commonly seen in COVID-19 patients [61]. In addition, COVID-19 may trigger a cytokine-mediated inflammatory response which leads to host-immune dysregulation predisposing to superimposed infections [61]. This may also explain the presence of EBV in brain tissue, which may be a consequence of viral reactivation in the setting of host-immune dysregulation-mediated and/or steroid exposure. It is possible that this patient may have first developed necrotizing hemorrhagic encephalitis in the temporal lobe as a consequence of cytokine-mediated inflammation or viral invasion, which was followed by superimposed invasive fungal infection and EBV reactivation in the setting of uncontrolled diabetes, glucocorticoid use, and immune dysregulation. SARS-CoV-2 PCR was negative in brain tissue and CSF, but a brain biopsy was done 4 weeks after the initial COVID-19 infection. Finally, the patient also had multiple supratentorial embolic infarcts and ischemic optic neuropathy, which was likely a consequence of hypercoagulability from COVID-19 infection rather than invasive mucormycosis, in the setting of recent COVID-19 infection and marked hyperfibrinogenemia [5,7]. Thus, this patient likely had the multifactorial disease, HE with superimposed infections and infarction. This case highlights the importance of having a high index of suspicion for secondary CNS infections in COVID-19 patients presenting with encephalopathy and abnormal neuroimaging, particularly in patients with underlying co-morbidities.

## 6. Conclusions

Hemorrhagic encephalitis is an uncommon but serious complication of COVID-19 and is multifactorial, mediated by cytokines, host inflammatory response, superimposed infections, hypoxemia, hypercoagulability, and possibly direct viral invasion. The clinical course may range from mild self-limiting illness to severe encephalitis with co-infection with other pathogens. Other comorbid conditions such as diabetes mellitus, chronic hypertension, and immunosuppression may predispose to severe disease and co-infections. A low threshold for neuroimaging, even with mild neuropsychiatric symptoms such as headaches, can help in early diagnosis and prompt management, potentially preventing further complications. Pathology may be needed for patients with disease refractory to optimum medical management and those that continue to worsen despite improvement in underlying COVID-19 infection. Further neuropathological studies are needed to explore the neuro-invasive potential of SARS-CoV-2.
